# Combined epidural-general anesthesia was associated with lower risk of postoperative complications in patients undergoing open abdominal surgery for pheochromocytoma: A retrospective cohort study

**DOI:** 10.1371/journal.pone.0192924

**Published:** 2018-02-21

**Authors:** Nan Li, Hao Kong, Shuang-Ling Li, Sai-Nan Zhu, Dong-Xin Wang

**Affiliations:** 1 Department of Anesthesiology and Critical Care Medicine, Peking University First Hospital, Beijing, China; 2 Department of Biostatistics, Peking University First Hospital, Beijing, China; Beth Israel Deaconess Medical Center, UNITED STATES

## Abstract

**Background:**

Current evidences show that regional anesthesia is associated with decreased risk of complications after major surgery. However, the effects of combined regional-general anesthesia remain controversial. The purpose of our study was to analyze the impact of anesthesia (combined epidural-general anesthesia vs. general anesthesia) on the risk of postoperative complications in patients undergoing open surgery for pheochromocytoma.

**Methods:**

This was a retrospective cohort study. 146 patients who underwent open surgery for pheochromocytoma (100 received combined epidural-general anesthesia and 46 received general anesthesia) in Peking University First Hospital from January 1, 2002 to December 31, 2015 were enrolled. The primary outcome was the occurrence of postoperative complications during hospital stay after surgery. Multivariate Logistic regression models were used to analyze the association between the choice of anesthetic method and the risk of postoperative complications.

**Results:**

17 (11.6%) patients developed complications during postoperative hospital stay. The incidence of postoperative complications was lower in patients with combined epidural-general anesthesia than in those with general anesthesia (6% [6/100] vs. 23.9% [11/46], P = 0.006). Multivariate Logistic regression analysis showed that use of combined epidural-general anesthesia (OR 0.219, 95% CI 0.065–0.741; P = 0.015) was associated with lower risk, whereas male gender (OR 5.213, 95% CI 1.283–21.177; P = 0.021) and perioperative blood transfusion (OR 25.879; 95% CI 3.130–213.961; P = 0.003) were associated with higher risk of postoperative complications.

**Conclusions:**

For patients undergoing open surgery for pheochromocytoma, use of combined epidural-general anesthesia may decrease the occurrence of postoperative complications.

## Introduction

Pheochromocytoma is a rare neuroendocrine tumor [1,2]. Surgical resection is the standard treatment. However, perioperative management is a great challenge for the anesthesiologists because of dramatic hemodynamic fluctuation and high risk of complications [3,4]. Laparoscopic surgery has the advantages of minimal invasiveness and high accuracy, and is increasingly used in the treatment of pheochromocytoma [5,6]. Indeed, Hattori et al. [7] reported an incidence of complications (≥ grade II on Clavien-Dindo classification) of 5.2% after laparoscopic surgery; whereas in the study of Elfenbein et al, [8] complications occurred in 18.8% of patients who underwent open abdominal surgery. Despite of these advantages, open surgery remains an option for patients with large and specially located (such as the inter-aorto-caval region) tumors [9].

Accumulating evidences show that, for patients undergoing major surgery, regional anesthesia is superior to general anesthesia in decreasing the incidence of postoperative complications (especially postoperative pulmonary complications) [10,11] and the requirement of blood transfusion after surgery [12]. However, whether combined regional-general anesthesia has advantage over simple general anesthesia remains controversial. We hypothesized that, for patients who planned to undergo open abdominal surgery for pheochromocytoma, combined epidural-general anesthesia might be better than general anesthesia alone regarding the incidence of postoperative complications. Unfortunately, few studies investigated this problem. The purpose of this retrospective study was to analyze the impact of anesthesia methods (combined epidural-general anesthesia vs. general anesthesia) on the risk of postoperative complications in patients who underwent open surgery for pheochromocytoma.

## Material and methods

This retrospective cohort study was conducted between May 25, 2016 and December 31, 2016. The study protocol was approved by the Clinical Research Ethics Committee of Peking University First Hospital (2016–1062). Because of the retrospective nature of the study and that all data of patients were collected from the medical records, the local Ethics Committee agreed to exempt written informed consent. The manuscript adhered to the applicable Equator guidelines (S1 Table).

### Patients

Potential participants were patients who underwent open abdominal surgery for pheochromocytoma with the diagnosis confirmed by postoperative pathologic examination in Peking University First Hospital from January 1, 2002 to December 31, 2015. Patients who met any of the following criteria were excluded: (1) age less than 18 years; (2) surgery was performed in the way other than open abdominal resection; (3) incomplete data collected from the medical record system.

### Anesthesia and analgesia

The choice of anesthesia and analgesia methods was made by the attending anesthesiologists. For patients who received combined epidural-general anesthesia, epidural puncture and catheterization was performed in the intervertebral space from T6 to T10 according to the region of surgery. Intraoperative epidural anesthesia was maintained with 1% lidocaine or 0.5% ropivacaine. For all patients, general anesthesia was induced with propofol, fentanyl or sufentanil, and rocuronium, and maintained with nitric oxide and sevoflurane inhalation, remifentanil or sufentanil infusion, and rocuronium or cisatracurium intermittent injection.

For patients with an epidural catheter, patient-controlled epidural analgesia was provided after surgery, which was established with 250 ml of 0.12% ropivacaine and 0.5 μg/ml sufentanil, programmed to deliver a 2 ml bolus with a lockout interval of 20 min and a background infusion of 4 ml/h. For those without an epidural catheter, patient-controlled intravenous analgesia was provided after surgery, which was established with 100 mL of 0.5 mg/ml morphine or 1.25 μg/ml sufentanil, programmed to deliver a 2 ml bolus with a lockout interval of 6–10 min and a background infusion of 1 ml/h.

All procedures were performed by qualified surgeons and anesthesiologists with clinical experiences of more than 10 years.

### Data collection

The list of patients with the diagnosis of pathologically confirmed pheochromocytoma from 2002 to 2015 was acquired through the electronic registry system of the Department of Urology. Patients’ data were then searched through the electronic medical record system of the hospital and eligible patients were identified according to the inclusion/exclusion criteria.

For included patients, perioperative data were collected. Preoperative data included demographic characteristics (gender, age, body mass index [BMI]), previous medical history, American Society of Anesthesiology (ASA) physical status classification, serum catecholamine concentrations, size and location of tumor, medical treatment, as well as heart rate and blood pressure before surgery. Intraoperative data included method and duration of anesthesia, duration of surgery, estimated blood loss, positive fluid balance, use of vasoactive drugs (vasopressors and antihypertensive drugs), and presence of hemodynamic fluctuations [13]. Postoperative data included use of vasopressors and their duration, postoperative analgesia, glucocorticoids administration, transfusion of blood products, duration of mechanical ventilation, length of stay in ICU and hospital, time to oral intake resumption, occurrence of postoperative complications, in-hospital mortality and medical care costs.

The primary endpoint was the incidence of postoperative complications during hospital stay after surgery. Postoperative complications were defined as newly onset medical conditions that were harmful to patients’ recovery and required therapeutic intervention, i.e., grade 2 or higher according to the Clavien-Dindo classification (S2 Table) [14]. For any diagnosed complication, the time of first diagnosis was also recorded. To ensure the accuracy of our database, two researchers collected the information of postoperative complications simultaneously and respectively. In case of a difference between the two researchers, final agreement was achieved by rechecking the records and full discussion with a senior physician (S1 Dataset).

### Statistical analysis

Patients’ data were analyzed according to the method of anesthesia (general vs. combined epidural-general anesthesia) and the development of postoperative complications. Numeric data with normal distribution were compared by independent samples t rest; numeric data with abnormal distribution or ranked data were compared by Mann-Whitney U test. Categorical data were compared by chi-square test or Fisher's exact test. Time-event data were analyzed by Kaplan-Meier estimator, with difference between groups compared by log- rank test. To identify independent risk factors of postoperative complications, variables with a P < 0.10 in univariate analyses were included in multivariate Logistic regression model (backward method). Two- sided P values of less than 0.05 were regarded as statistically significant. All statistical analyses were performed with the SPSS statistical package version 14.0 (SPSS Inc, Chicago, IL, USA).

## Results

**Fig 1:**
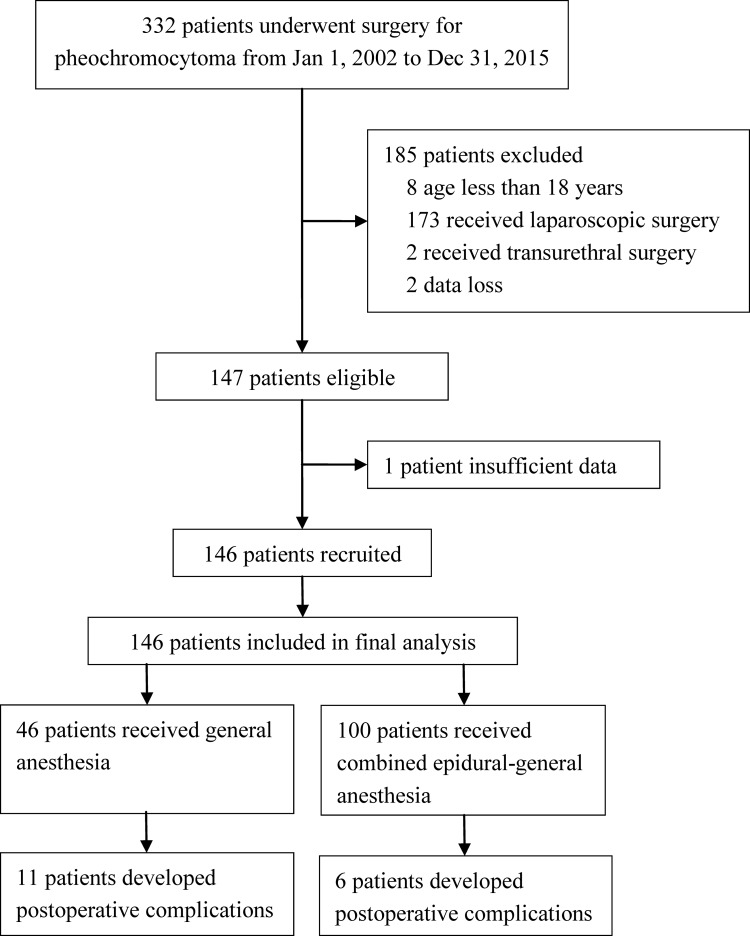
Flow diagram of the study.

From January 1, 2002 to December 31, 2015, 332 patients underwent surgery for pheochromocytoma; among them 147 met the inclusion/exclusion criteria, 146 were included in final analysis (Fig 1). Among the enrolled patients, 46 (31.5%) received general anesthesia and patient-controlled intravenous analgesia after surgery (including 4 patients who had failed epidural puncture and catheterization), the other 100 (68.5%) received combined epidural-general anesthesia and patient-controlled epidural analgesia after surgery; 17 (11.6%) developed complications during postoperative hospital stay (the incidence of postoperative complications after laparoscopic procedures was 5.8% [10/173]). Baseline and perioperative data were listed in Tables 1 and 2 (also see S2 Table).

**Table 1:** Preoperative variables.

Variable	All patients (n = 146)	General anesthesia (n = 46)	Combined epidural-general anesthesia (n = 100)	P value	Without postoperative complications (n = 129)	With postoperative complications (n = 17)	P value
Age (years)	45±15	52±14	42±14	< 0.001	45±14	48±20	0.434
Gender (male)	68 (46.6%)	28 (60.9%)	40 (40.0%)	0.019	54 (41.9%)	14 (82.4%)	0.002
BMI (kg/m2)	23.1±3.8	24.1±3.6	22.6±3.8	0.029	23.0±3.8	23.7±3.6	0.488
Preoperative comorbidity							
Diabetes mellitus	26 (17.8%)	10 (21.7%)	16 (16.0%)	0.400	23 (17.8%)	3 (17.6%)	0.530
Coronary heart disease	6 (4.1%)	2 (4.3%)	4 (4.0%)	> 0.999	5 (3.9%)	1 (5.9%)	0.531
Stroke	9 (6.2%)	4 (8.7%)	5 (5.0%)	0.463	7 (5.4%)	2 (11.8%)	0.282
ASA classification				0.036			0.556
1–2	111 (76.0%)	40 (87.0%)	71 (71.0%)		99 (76.7%)	12 (70.6%)	
3–4	35 (24.0%)	6 (13.0%)	29 (29.0%)		30 (23.3%)	5 (29.4%)	
Preoperative Hb (g/L)	133±17	134±18	133±17	0.642	133±18	134±17	0.939
Concentration of serum catecholamine ^*a*^							
Dopamine (pmol/L)	0.18 (0.07, 0.38)	0.11 (0.08, 0.36)	0.31 (0.05, 0.39)	0.739	0.24 (0.07, 0.38)	0.10 (0.09, 0.11)	0.370
Norepinephrine (pmol/L)	10.5 (2.4, 27.1)	6.9 (2.7, 20.8)	12.3 (2.3, 31.4)	0.409	8.7 (2.4,25.8)	13.1 (5.2, 24.8)	0.620
Epinephrine (pmol/L)	0.68 (0.43, 1.46)	0.62 (0.28, 1.81)	0.71 (0.43, 1.46)	0.521	0.62 (0.19, 2.51)	0.96 (0.75, 3.36)	0.293
Maximal diameter of tumor (cm) ^*b*^	7.2±3.7	6.9±3.8	7.3±3.7	0.576	7.0±3.6	8.4±4.2	0.143
Ectopic tumor ^*c*^	43 (29.5%)	11 (23.9%)	32 (32.0%)	0.319	39 (30.2%)	4 (23.5%)	0.569
Preoperative medication							
α receptor antagonist ^*d*^	133 (91.1%)	41 (89.1%)	92 (92.0%)	0.548	117 (90.7%)	16 (94.1%)	> 0.999
β receptor antagonist	38 (26.0%)	8 (17.4%)	30 (30.0%)	0.107	34 (26.4%)	4 (23.5%)	> 0.999
Calcium channel blocker	44 (30.1%)	12 (26.1%)	32 (32.0%)	0.469	39 (30.2%)	5 (29.4%)	0.945
Combined antihypertensives	57 (39.0%)	18 (39.1%)	39 (39.0%)	0.988	49 (38.0%)	8 (47.1%)	0.471
Intravenous fluid therapy ^*e*^	66 (45.2%)	19 (41.3%)	47 (47.0%)	0.521	59 (45.7%)	7 (41.2%)	0.723
Preoperative SBP (mmHg) ^*f*^	127±15	125±16	127±15	0.417	126±15	133±16	0.070
Preoperative DBP (mmHg) ^*f*^	79±12	77±10	80±12	0.053	79±12	83±11	0.164
Preoperative HR (bpm) ^*f*^	76±9	76±7	76±10	0.977	76±9	77±6	0.754

Data were presented as mean ± standard deviation, number of patients (percentage), or median (interquartile range), unless otherwise indicated.

BMI, Body Mass Index; ASA, American Society of Anesthesiologists; SBP, Systolic blood pressure; DBP, Diastolic blood pressure; HR, Heart rate; Hb, hemoglobin.

^*a*^ Measured in calm state before admission

^*b*^ According to postoperative pathologic examination results

^*c*^ Pheochromocytoma situated outside adrenal gland

^*d*^ Several patients did not receive α receptor antagonist therapy due to normal blood pressure and serum catecholamine concentrations before surgery. Diagnosis of pheochromocytoma was confirmed by postoperative pathologic examination

^*e*^ Intravenous infusion of crystalloid and/or colloid after admission

^*f*^ Measured in the ward on the day before surgery.

**Table 2:** Intra- and postoperative variables.

Variable	All patients (n = 146)	General anesthesia (n = 46)	Combined epidural-general anesthesia (n = 100)	P value	Without postoperative complications (n = 129)	With postoperative complications (n = 17)	P value
Period of surgery ^*a*^				0.004			0.121
2002–2006	43 (29.5%)	5 (10.9%)	38 (38.0%)		39 (30.2%)	4 (23.5%)	
2007–2011	57 (39.0%)	23 (50.0%)	34 (34.0%)		53 (41.1%)	4 (23.5%)	
2012–2015	46 (31.5%)	18 (39.1%)	28 (28.0%)		37 (28.7%)	9 (52.9%)	
Duration of anesthesia (min)	301 ± 121	301 ± 129	302 ± 118	0.985	289 ± 107	395 ± 174	0.025
Duration of surgery (min)	218 ± 114	209 ± 114	221 ± 114	0.559	207 ± 102	294 ± 163	0.039
Intraoperative minimal Hb (g/L)	98 ± 21	99 ± 18	97 ± 22	0.601	99 ± 21	89 ± 21	0.097
Intraoperative management							
Estimated blood loss (ml)	500 (100, 1200)	450 (100, 1000)	500 (200, 1200)	0.245	350 (100, 1000)	1500 (800, 3000)	0.002
Positive fluid balance (ml)	3000 (2100, 4450)	2525 (1400, 3600)	3100 (2400, 4550)	0.007	3000 (2075, 4400)	3100 (2300, 6050)	0.415
Combined antihypertensives ^*b*^	99 (67.8%)	31 (67.4%)	68 (68.0%)	0.942	87 (67.4%)	12 (70.6%)	0.794
Combined vasopressors ^*c*^	45 (30.8%)	10 (21.7%)	35 (35.0%)	0.107	37 (28.7%)	8 (47.1%)	0.123
Hemodynamic fluctuations ^*d*^	133 (91.1%)	40 (87.0%)	93 (93.0%)	0.347	119 (92.2%)	14 (82.4%)	0.179
Postoperative management							
Infusion of vasopressors	41 (28.1%)	6 (13.0%)	35 (35.0%)	0.006	36 (27.9%)	5 (29.4%)	>0.999
Duration of vasopressor (hr) ^*e*^	7.4 (3.6, 11.3)	3.8 (0.0, 8.1)	9.1 (3.9, 14.3)	0.110	5.7 (2.6, 8.9)	20.3 (0.0, 42.3)	0.042
Combined epidural-general anesthesia ^*f*^	100 (68.5%)	0 (0.0%)	100 (68.5%)	—	94 (72.9%)	6 (35.3%)	0.002
Intraoperative glucocorticoids ^*g*^	119 (81.5%)	41 (89.1%)	78 (78.0%)	0.108	103 (79.8%)	16 (94.1%)	0.199
Postoperative glucocorticoids ^*g*^	54 (37.0%)	13 (28.3%)	41 (41.0%)	0.139	44 (34.1%)	10 (58.8%)	0.047
Perioperative blood transfusion ^*h*^	69 (47.3%)	23 (50.0%)	46 (46.0%)	0.653	53 (41.1%)	16 (94.1%)	<0.001
Postoperative ICU admission	114 (78.1%)	33 (71.7%)	81 (81.0%)	0.209	99 (76.7%)	15 (88.2%)	0.364
Use of MV	77 (52.7%)	22 (47.8%)	55 (55.0%)	0.420	65 (50.4%)	12 (70.6%)	0.117
Duration of MV (hr) ^*i*^	3.0 (1.8, 4.3)	3.5 (1.4, 5.5)	2.8 (1.2, 4.5)	0.748	2.0 (1.4, 2.7)	10.6 (1.3, 19.9)	0.002
ICU stay (day) ^*j*^	1.8 (1.5, 2.0)	2.0 (1.6, 2.5)	1.6 (1.3, 2.0)	0.156	1.5 (1.4, 1.7)	3.3 (1.9, 4.6)	<0.001

Data were presented as mean ± standard deviation, number of patients (percentage), or median (interquartile range), unless otherwise indicated.

Hb, hemoglobin; ICU, intensive care unit; MV, mechanical ventilation.

^*a*^ See more detail in S1 Text

^*b*^ Combined use of two or more intravenous antihypertensive drugs, including phentolamine, urapidil, nicardipine and esmolol

^*c*^ Combined use of two or more intravenous vasopressors, including ephedrine, phenylephrine, norepinephrine, epinephrine and dopamine

^*d*^ Defined when met any of the following criteria: (1) Systolic blood pressure ≥ 200 mmHg or increased to more than 30% above baseline; (2) Systolic blood pressure ≤ 90 mm Hg; (4) Heart rate ≥ 110 bpm; (5) Heart rate ≤ 50 bpm

^*e*^ Result of patients requiring intravenous vasopressor infusion. Data were analyzed by Kaplan-Meier analysis and compared by log-rank test; results were presented as average (95% confidence interval)

^*f*^ These patients received postoperative patient-controlled epidural analgesia (PCEA)

^*g*^ Including dexamethasone, hydrocortisone, methylprednisolone

^*h*^ Intra- and/or postoperative blood products transfusion, including packed red blood cell, plasma and platelet

^*i*^ Results of patients requiring postoperative mechanical ventilation. Data were analyzed by Kaplan-Meier analysis and compared by log-rank test; results were presented as average (95% confidence interval)

^*j*^ Result of patients admitted to ICU. Data were analyzed by Kaplan-Meier analysis and compared by log-rank test; results were presented as average (95% confidence interval).

**Fig 2:**
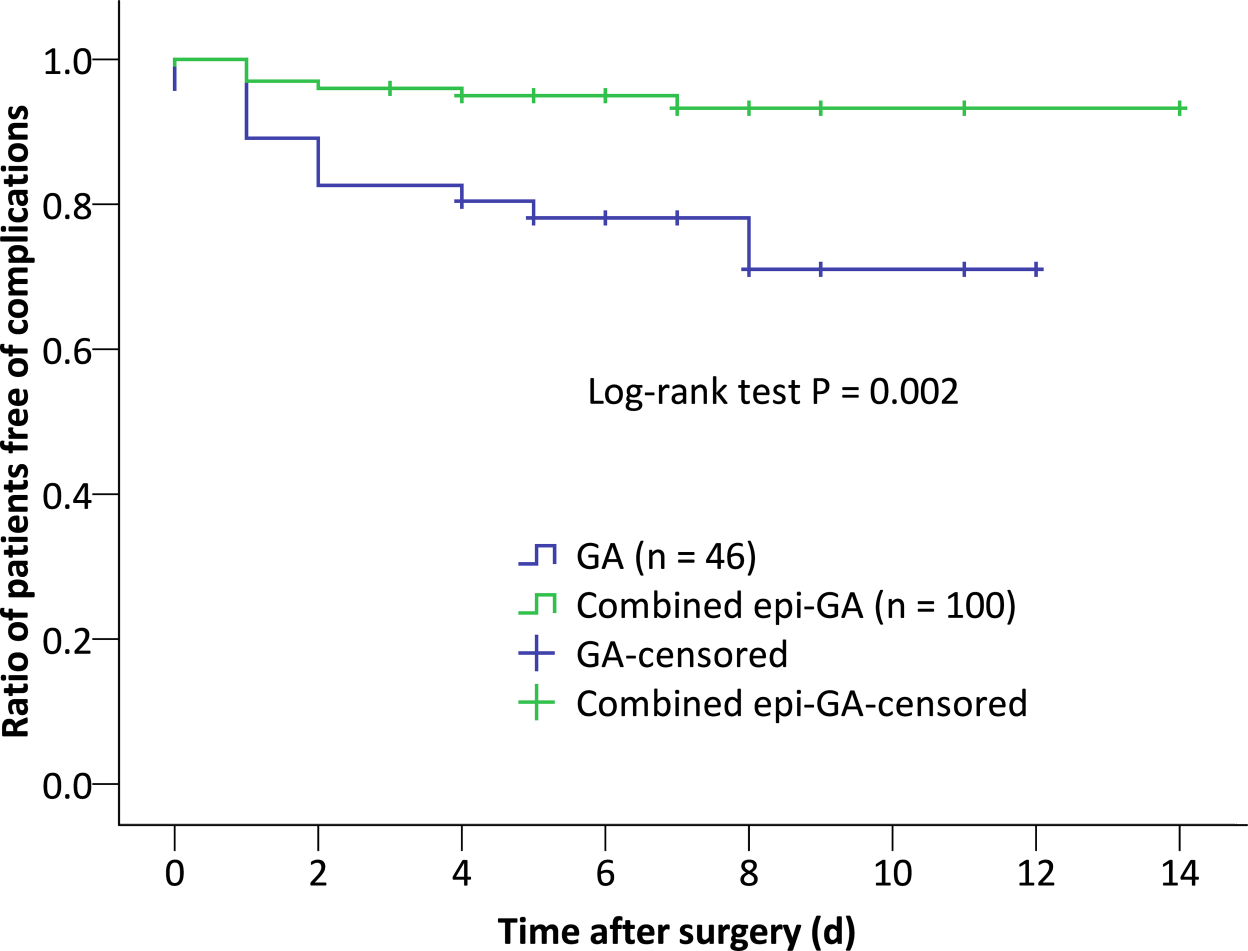
Ratio of patients free of postoperative complications. Postoperative complications were defined as grade II or higher on the Clavien-Dindo classification. PC = postoperative complications.

The incidence of postoperative complications was lower in patients with combined epidural-general anesthesia than in those with general anesthesia (6.0% [6/100] vs. 23.9% [11/46], P = 0.006). Furthermore, the number of postoperative complications (Clavien-Dindo grade I or higher) was less (P = 0.006) and the severity of postoperative complications was less severe (P = 0.017) in patients with combined epidural-general anesthesia than in those with general anesthesia (Table 3, Fig 2, S2 Table).

**Table 3:** Postoperative outcomes.

Variable	All patients (n = 146)	General anesthesia (n = 46)	Combined epidural-general anesthesia (n = 100)	P value
Number of postoperative complications ^*a*^	0 (0, 2)	0 (0, 2)	0 (0, 2)	0.006
Severity of postoperative complications ^*b*^				0.017
I	3 (2.1%)	2 (4.3%)	1 (1.0%)	
II	10 (6.8%)	6 (13.0%)	4 (4.0%)	
IIIa	4 (2.7%)	2 (4.3%)	2 (2.0%)	
IIIb	2 (1.4%)	2 (4.3%)	0 (0.0%)	
IV	0 (0.0%)	0 (0.0%)	0 (0.0%)	
V	1 (0.7%)	1 (2.2%)	0 (0.0%)	
Occurrence of postoperative complications ^*c*^	17 (11.6%)	11 (23.9%)	6 (6.0%)	0.006
Occurrence of postoperative pulmonary complications ^*c*^	9 (6.2%)	5 (10.9%)	4 (4.0%)	0.141
Resumption of oral intake after surgery (day) ^*d*^	3.2 (2.9, 3.6)	3.4 (2.7, 4.1)	3.1 (2.8, 3.6)	0.497
Postoperative hospital stay (day) ^*d*^	7.5 (6.8, 8.1)	8.1 (6.4, 9.8)	7.2 (6.6, 7.7)	0.156
Postoperative in-hospital mortality	2 (1.4%)	1 (2.2%)	1 (1.0%)	0.532
Total medical cost (10,000 yuan) ^*e*^	5.0 (4.2, 8.6)	5.1 (4.4, 5.8)	5.0 (4.2, 8.6)	0.344

Data were presented as number of patients (percentage), or median (95% confidence interval), unless otherwise indicated.

^*a*^ Complications of grade I or higher on Clavien-Dindo classification; results were presented as median (full range)

^*b*^ In patients with two or more complications, the grade of the most severe complication was recorded. Data were compared by Mann-Whitney U test

^*c*^ Complication of grade II or higher

^*d*^ Data were analyzed by Kaplan-Meier analysis and compared by log-rank test; results were presented as average (95% confidence interval)

^*e*^ Data were presented as median (interquartile range).

Univariate analyses identified 8 factors that might be associated with the occurrence of postoperative complications (P < 0.10), including gender, preoperative systolic blood pressure, method and duration of anesthesia, duration of surgery, estimated blood loss during surgery, postoperative glucocorticoids administration and perioperative blood transfusion. After excluding duration of anesthesia (with duration of surgery) and estimated blood loss during surgery (with perioperative blood transfusion) because of collinearity, other factors were included in the multivariate Logistic regression model (backward). 3 factors were identified to be independently associated the occurrence of postoperative complications; among them male gender (OR 5.213; 95% CI 1.283–21.177; P = 0.021) and perioperative blood transfusion (OR 25.879; 95% CI 3.130–213.961; P = 0.003) were associated with higher risk, whereas combined epidural-general anesthesia (OR 0.219; 95% CI 0.065–0.741; P = 0.015) was associated with lower risk (Table 4, also see S1 Text).

**Table 4:** Risk factors of postoperative complications.

Variable	Univariate Logistic model		Multivariate Logistic model ^*a*^	
	OR (95% CI)	P value	OR (95% CI)	P value
Male gender	6.481 (1.775–23.666)	0.005	5.213 (1.283–21.177)	0.021
Preoperative systolic blood pressure (every 10 mmHg increase) ^*b*^	1.360 (0.972–1.902)	0.072	—	—
Duration of surgery (every 1 hour increase)	1.429 (1.122–1.820)	0.004	—	—
Perioperative blood transfusion ^*c*^	22.943 (2.952–178.312)	0.003	25.879 (3.130–213.961)	0.003
Combined epidural-general anesthesia	0.203 (0.070–0.591)	0.003	0.219 (0.065–0.741)	0.015
Postoperative glucocorticoids administration ^*d*^	2.760 (0.983–7.747)	0.054	—	—

OR, odds ratio; CI, confidence interval.

^*a*^ Variables with P < 0.10 in univariate analyses were included in multivariate Logistic regression model (Backward: LR). Also see S1 Text.

^*b*^ Measured in the ward on the day before surgery

^*c*^ Intra- and/or postoperative transfusion of blood products, including packed red blood cell, plasma and platelet

^*d*^ Including dexamethasone, hydrocortisone, or methylprednisolone.

## Discussion

Results of this retrospective study showed that, in patients undergoing open abdominal surgery for pheochromocytoma under general anesthesia, combined use of epidural anesthesia (and postoperative epidural analgesia) was associated with lower risk of postoperative complications; furthermore, combined use of epidural anesthesia was associated with less severe complications. Considering the relatively uncommon nature of the disease and the lack of evidence of anesthetic impact on patients’ outcomes, our results provided novel and valuable information to anesthesia practitioners.

In the present study, postoperative complications were defined as newly occurred conditions that required therapeutic intervention (i.e., grade II or higher on the Clavien-Dindo classification), in order to avoid ambiguity. In our patients, 11.6% developed postoperative complications, similar to the prospective results of Niren et al. [9] who reported a 10% incidence in a similar patient population. And, in our patients who developed postoperative complications, 52.9% (9/17) had their complications originated from the respiratory system, result in a 6.2% incidence of postoperative pulmonary complications. This was in line with the 7.2% incidence of pulmonary complications after open gastrointestinal surgery reported by Canet et al. [15] Our results showed that the incidence of overall postoperative complications was lower in patients with combined epidural-general anesthesia than in those with general anesthesia.

The beneficial effects of neuraxial blockade on the occurrence of postoperative complications have been reported previously. For example, use of neuraxial analgesia reduced the risk of major nonsurgical complications in patients after abdominal aortic surgery [16]. When compared with general anesthesia, use of regional anesthesia was associated with lower incidences of composite and, especially, pulmonary morbidities, in patients with chronic obstructive pulmonary disease [11]. For patients undergoing colorectal surgery, combined use of thoracic epidural anesthesia/analgesia improved pain control, facilitated early mobilization and recovery of gut function, and reduced gastrointestinal complication [17–19]. A meta-analysis also showed that use of epidural analgesia reduced pulmonary complications after abdominal and thoracic surgery, probably due to earlier mobilization, reduced opioid consumption, and improved cough [20]. In a recent study, use of perioperative thoracic epidural analgesia reduced the occurrence of major adverse cardiac events (including deep venous thromboembolism) after major abdominal cancer surgery in patients suffering from coronary artery disease [21]. For the first time, our results showed that combined use of epidural anesthesia/analgesia was associated with decreased risk of postoperative complications in patients undergoing open abdominal surgery for pheochromocytoma. This needs further demonstration by randomized control trials.

The mechanisms by which combined epidural-general anesthesia (and epidural analgesia) provides protection for perioperative patients may include the following. Firstly, it is more effective in relieving acute pain and pain-related harmful effects [22]. Secondly, thoracic epidural anesthesia may improve the balance between myocardial oxygen consumption and supply, and relieve gut injury [23]. Thirdly, it relieves the over activation of neuroendocrine, metabolic and inflammatory response after surgery [24]. Lastly, epidural blockade in addition to general anesthesia may prevent the fluctuation of hormone levels in patients undergoing adrenalectomy for adrenal functional tumors [25].

In the present study, perioperative blood transfusion was another factor associated with increased risk of postoperative complications. In line with our results, Venkat et al. [26] reported that intraoperative transfusion was incrementally associated with significant morbidity and mortality after adrenalectomy. Similar phenomenon was also confirmed in other surgical populations, such as those undergoing colorectal cancer surgery [27], hepatectomy [28], and gastrectomy [29]. This finding can be explained by the following reasons. On one hand, requirement of perioperative transfusion usually indicates a more extensive disease and surgical trauma, and therefore, a more severe influence on patients. On the other hand, as an allograft tissue, blood products per se can produce harmful effects by inhibiting immune function [30,31]. A recent study of Kim et al.[32] found that transfusion of "older" blood might contribute to a higher risk of postoperative morbidity when compared to “fresh” blood. In our results, male gender was also a risk factor of postoperative complications. Interestingly, there were studies revealed that male gender was associated with longer duration of surgery [33], higher risk of postoperative deep venous thrombosis [34], and increased mortality [35]. The association between gender and patients’ outcome after surgery for pheochromocytoma needs to be further evaluated.

Except the retrospective nature, there were some other limitations in our study. Firstly, patients’ data were collected until hospital discharge. In a prospective study, John et al.[36] found that about one-third of complications occurred between discharge and 30 days after surgery. Our results might have underestimated the incidence of postoperative complications. Secondly, given the long duration of this study, many innovations or new treatments had been introduced during this period as part of the practical management, thus might confound the results. However, inclusion of surgical period in the multivariate logistic model did not change the results (see S1 Text). Finally, as a single-center study, the generalizability of our conclusions might be limited.

## Conclusions

Our results showed that, in patients scheduled to undergo open abdominal surgery for pheochromocytoma, combined epidural-general anesthesia (and epidural analgesia after surgery) was associated with decreased risk of postoperative complications when compared with general anesthesia alone. Prospective randomized control trials are needed to verify these findings.

## Supporting information

S1 Table STROBE checklist. (DOC)

S2 Table Occurrence of postoperative complications. (DOCX)

S1 Dataset Relevant data underlying the main results. (XLSX)

S1 Text Sensitivity analysis by splitting the whole study period into four- or five-year sessions. (DOCX)
