# Supplementary material for: Combined epidural-general anesthesia was associated with lower risk of postoperative complications in patients undergoing open abdominal surgery for pheochromocytoma: A retrospective cohort study
Source: PLoS One. 2018 Feb 21;13(2):e0192924. doi: 10.1371/journal.pone.0192924 (PMC5821342; doi:10.1371/journal.pone.0192924)
Supplement: S2 Table — (DOCX) [file pone.0192924.s002.docx]

S2 Table. Occurrence of postoperative complications

| Complications | All patients (n=146) | General anesthesia (n=46) | Combined epidural-general anesthesia (n=100) |
| --- | --- | --- | --- |
| Pulmonary complications | 9 | 5 | 4 |
| Pulmonary infection *^a^* | 4 | 2 | 2 |
| Pleural effusion *^b^* | 3 | 1 | 2 |
| Atelectasis *^c^* | 2 | 2 | 0 |
| Respiratory failure *^d^* | 0 | 0 | 0 |
| Surgical bleeding *^e^* | 2 | 2 | 0 |
| New onset arrhythmia *^f^* | 1 | 1 | 0 |
| Acute myocardial infarction *^g^* | 0 | 0 | 0 |
| Congestive heart failure *^h^* | 0 | 0 | 0 |
| Stroke *^i^* | 0 | 0 | 0 |
| Ileus *^j^* | 1 | 1 | 0 |
| Liver injury *^k^* | 0 | 0 | 0 |
| Wound infection *^l^* | 0 | 0 | 0 |
| Urinary tract infection *^m^* | 0 | 0 | 0 |
| Severe sepsis *^n^* | 0 | 0 | 0 |
| Acute kidney injury *^o^* | 5 | 3 | 2 |
| Stage 1 | 3 | 1 | 2 |
| Stage 2 | 1 | 1 | 0 |
| Stage 3/renal failure | 1 | 1 | 0 |
| Digestive tract bleeding *^p^* | 0 | 0 | 0 |
| Venous thromboembolism | 3 | 1 | 2 |
| Pulmonary embolism *^q^* | 1 | 0 | 1 |
| Deep venous thrombosis *^r^* | 2 | 1 | 1 |

*^a^* Presence of at least one of the following manifestations (increased or color-changed sputum, new or changed pulmonary infiltrates, fever, leukocyte count > 12,000/mm^3^) and required antibiotic therapy;

*^b^* Confirmed by chest X-ray or ultrasound examination and required therapeutic intervention (drainage, aspiration, and/or diuresis after albumin administration); d

*^c^* Confirmed by chest X-ray examination, with or without oxygen desaturation, and required therapeutic intervention (oxygenation inhalation, physical therapy, and/or mechanical ventilation);

*^d^* Presence of the following manifestations (PaO_2_ <60 mmHg on room air, ratio of PaO_2_ to inspired oxygen fraction <300, or oxygen saturation <90%) and required therapeutic intervention (oxygen therapy or mechanical ventilation) for more than 24 hours;

*^e^* Bleeding after surgery that required secondary surgical hemostasis;

*^f^* New onset atrial fibrillation or paroxysmal supraventricular tachycardia that necessitated medical treatment;

*^g^* Concentration of cardiac troponin I exceed the diagnostic criteria for myocardial infarction as well as new Q waves (lasts for 0.03 s) or continuous (4 days) abnormal ST-T segment;

*^h^* Dyspnea and elevated brain natriuretic peptide level necessitating diuresis and noninvasive mechanical ventilation;

*^i^* Persisted new focal neurologic deficit and confirmed by neurologic imaging;

*^j^* Lack of bowel movement, flatulence, and requirement of parenteral nutrition for more than 1 week after surgery;

*^k^* Elevation of serum transaminase level above 3 times the upper limit, excluded myocardial and skeletal muscle injury;

*^l^* Pus expressed from the incision, and bacteria cultured from the pus;

*^m^* Confirmed by urinalysis and urine culture and necessitated antibiotic therapy;

*^n^* Two or more criteria of systemic inflammatory response syndrome, with known infection and new onset dysfunction of at least one system;

*^o^* Diagnosed according to KDIGO criteria using serum creatinine level and urine output volume after surgery;

*^p^* Decrease of hemoglobin level combined with positive gastrointestinal occult blood test results that required treatment;

*^q^* Pulmonary embolism: confirmed by CTPA;

*^r^* Deep venous thrombosis: confirmed by deep venous ultrasonography.
