# Supplementary material for: Combined epidural-general anesthesia was associated with lower risk of postoperative complications in patients undergoing open abdominal surgery for pheochromocytoma: A retrospective cohort study
Source: PLoS One. 2018 Feb 21;13(2):e0192924. doi: 10.1371/journal.pone.0192924 (PMC5821342; doi:10.1371/journal.pone.0192924)
Supplement: S1 Text — (DOCX) [file pone.0192924.s004.docx]

**S1 Text. Sensitivity analysis by splitting the whole study period into four- or five-year sessions**

Because of the low incidence of pheochromocytoma, our study covered a relative long period (from 2002 to 2015), during which changes of perioperative care happened. The figure below showed that the number of patients undergoing pheochromocytoma surgery (including both open and laparoscopic surgeries) increased, whereas the proportions of patients undergoing open surgery and combined epidural-general anesthesia decreased during this period (also see Table 2). We divided the whole period to 3 consecutive sessions, i.e., 2002-2006, 2007-2011, and 2012-2015. Univariate regression analysis showed that the session of surgery was not associated with the risk of postoperative complications (P=0.130). Even if the session of surgery was included in the multivariate model, it was not significantly associated with the risk of postoperative complications.
